# Comparing the effects of endometrial injury in the luteal phase and follicular phase on *in vitro* fertilization treatment outcomes

**DOI:** 10.3389/fendo.2022.1004265

**Published:** 2022-10-17

**Authors:** Yang Wang, Zhiqin Bu, Linli Hu

**Affiliations:** Reproductive Medical Center, the First Affiliated Hospital of Zhengzhou University, Zhengzhou, China

**Keywords:** endometrial injury, luteal phase, follicular phase, *in vitro* fertilization, pregnancy outcome

## Abstract

**Background:**

Several studies have shown that endometrial injury improves clinical pregnancy outcomes in patients undergoing *in vitro* fertilization/intracytoplasmic sperm injection (IVF/ICSI) treatment with a history of implantation failure. However, endometrial injury can be performed in the follicular phase (FP) followed by embryo transfer in the same menstrual cycle or in the luteal phase (LP) before the embryo transfer cycle.

**Method:**

This prospective cohort study was conducted from January 2015 to September 2021, and a total of 487 patients were included. All included patients had a history of a failed implantation cycle. They were divided into two groups: the FP group (*N* = 330), in which endometrial injury was performed on menstrual day 3-5, and the LP group (*N* = 157), in which endometrial injury was performed in the cycle preceding embryo transfer 7 days after ovulation.

**Results:**

First, in unselected patients, the implantation rate and clinical pregnancy rate were comparable between the LP and FP groups. However, in patients with a history of ≥ 2 failed transfer cycles, the implantation rate was significantly higher in the LP group than in FP group (43.09% versus 33.33%, *P* = 0.03). Moreover, the clinical pregnancy rate was also significantly higher in patients in the LP group than in patients in the FP group (60.17% versus 46.15%, *P*=0.02). In addition, logistic regression analysis showed that endometrial injury in the LP group was an independent factor affecting clinical pregnancy outcome in patients with a history of ≥ 2 failed transfer cycles (aOR = 2.05, 90% CI:1.22-3.47, *P*=0.01).

**Conclusion:**

Endometrial injury improves pregnancy outcomes when performed in the luteal phase compared with the follicular phase in patients with a history of ≥ 2 failed transfer cycles but not in unselected patients.

## Introduction

In the past few years, assisted reproductive techniques (ART), which mainly includes *in vitro* fertilization/intracytoplasmic sperm injection (IVF/ICSI), has evolved rapidly. However, embryo implantation failure is still a substantial problem that places considerable pressure on both patients and clinicians. For some young patients, even if high-quality embryos are transferred, implantation is still difficult. Since embryo quality and endometrial receptivity are two main factors for a successful pregnancy during IVF/ICSI, a possible explanation for this phenomenon is that these failed cycles have poor endometrial receptivity.

Endometrial injury, which is also known as endometrial scratching, is usually defined as intentional trauma to the endometrium by a biopsy catheter (1, 2). Recently, several studies have reported that endometrial injury improves clinical pregnancy outcomes in patients undergoing IVF/ICSI treatment with a history of implantation failure (3–5). Other clinical studies, as well as several meta analyses, did not show any difference in regard to pregnancy outcomes between women who underwent endometrial injury and those who did not (6, 7). In our center, it has been more than 10 years since the first endometrial injury was performed in patients with implantation failure. Our data confirm that endometrial injury improves pregnancy outcomes for women undergoing repeated IVF cycles.

In previous studies, endometrial injury was performed in either the follicular phase (FP) followed by embryo transfer in the same menstrual cycle or in the luteal phase (LP) before the embryo transfer cycle. A recent study showed that the timing of endometrial injury can make a major difference for the final pregnancy outcome (8). However, only few RCTs with a small sample size compared the difference in pregnancy outcomes in patients with endometrial injury in the FP and LP (9).

Thus, the current prospective observational study aimed to explore the impact of endometrial injury (FP vs. LP) on pregnancy outcomes in women undergoing frozen thawed embryo transfer (FET) cycles, especially in patients with different histories of implantation failure.

## Materials and methods

A total of 487 women with a history of failed implantation cycles were included into this study. This study was approved by the Medical Ethics Committee Board of First Affiliated Hospital of Zhengzhou University. All patients included were fully informed and signed written informed consent to allow us using their data for publication. Exclusion criteria were: Uterine anomaly; uterine adhesion, oocyte donation cycles; Pre-implantation Genetic Testing (PGT) cycles.

### Treatment protocol

According to our Standard Operating Procedure, preparation of endometrium in frozen thawed embryo transfer was mainly divided into natural cycles (NCs) and artificial (estrogen-progesterone [EP]) cycles. Briefly, for NCs, patients were advised to perform ultrasonic evaluation starting on day 7-9 of the menstrual cycle. Blood samples were obtained for progesterone and LH levels once leading follicle reached 14 mm. After ovulation, embryos were transferred 3 or 5 days later, based on the stage of embryos cryopreserved. For EP cycles, low dose of oral estradiol (1 mg [progynova]; Bayer, Germany) was begun twice a day on cycle day 3 for the first 4 days. This dose was adjusted based on endometrial thickness every 4 days. Around 12-14 days later, progesterone in oil was added. Embryo transfer was performed 3 or 5 days later. Endometrial thickness was measured on the day of progesterone admission. All patients included were transferred with 2 cleavage embryos or 1 blastocyst according to Standard Operating Procedure in our center. To assess IVF outcome, serum human chorionic gonadotropin (hCG) was measured 14 days after embryo transfer. Clinical pregnancy was confirmed by ultrasound 5 weeks after embryo transfer.

### Endometrial injury

For patients in FP group, endometrial injury was performed on menstrual day 3-5. Embryos were transferred approximately 2 weeks after injury in the same cycle. Patients in the LP group underwent endometrial injury in the cycle preceding embryo transfer. Luteal phase was defined as 7 days after ovulation. Endometrial injury was performed by the same physician with Pipelle (Disposable Endometrium Suction Tube, S-3.2; Nuode Medical, Jiangxi, China). The standard procedure was as follows: the patient was placed in the bladder lithotomy position under the guidance of abdominal ultrasound, and the Pipelle was gently probed into the uterine cavity. Endometrial tissue was taken from the Pipelle with little negative pressure.

First, patient basic parameters and pregnancy outcomes were compared between patients in the FP and LP groups. In addition, these parameters were compared in a special group of patients with a history of ≥ 2 failed transfer cycles. Finally, adjusted logistic regression analysis was used to explore potential factors affecting clinical pregnancy rate in unselected patients and patients with ≥ 2 failed transfer cycles.

Several definitions in the current study were as follows: Implantation rate was the number of sacs detected on ultrasound divided by the number of embryos transferred. Clinical pregnancy was defined as the presence of at least one gestational sac on ultrasound at 5 weeks after embryo transfer. Ectopic pregnancy was defined as in previous study (10). The early miscarriage rate was defined as the number of miscarriages before 20 weeks divided by the number of women with a positive pregnancy test.

Data were analyzed using SPSS (Statistical Package for Social Science, SPSS Inc, Chicago, IL, USA) 17.0 software. The continuous data were shown as Mean ± Standard Deviation, and Student T-test was used for comparison between groups. The countable data were compared using Chi-square test. In adjusted logistic regression analysis, confounding factors included were female age, endometrial thickness on day of progesterone administration, stage of embryos transferred, endometrial preparation protocol, and time of endometrial injury (LP versus FP). *P* < 0.05 was considered statistically significant.

## Results

From January 2015 to September 2021, a total of 487 patients (157 luteal phase and 330 follicular phase) were included into this study. As shown in **Table 1**, there were no differences between these two groups with regard to female age, body mass index, infertility duration, infertility diagnosis, stage of embryos transferred, or endometrial preparation protocol. However, the endometrial thickness on the progesterone administration day was significantly thicker in the LP group (11.36 mm versus 10.23 mm; *P* = 0.01). The clinical pregnancy outcomes in patients undergoing endometrial injury in the two groups are also shown in **Table 1**. No significant differences regarding the implantation rate and clinical pregnancy rate were detected in these two groups, even though both parameters were higher in the LP group. Moreover, both ectopic pregnancy and early miscarriage rates were lower in the LP group, and the difference was not statistically significant.

**Table 1:** Basic parameters and pregnancy outcomes in patients undergoing endometrial injury in two groups.

	Luteal phase N=157	Follicular phase N=330	*P* value
Age (year)	31.71 ± 4.63	31.54 ± 5.01	0.34
Infertility duration (year)	4.36 ± 3.28	4.66 ± 3.67	0.59
BMI (kg/m^2^)	22.43 ± 2.67	22.74 ± 3.43	0.46
Infertility diagnosis			
* Tubal factor*	44.59% (70/157)	40.91% (135/330)	0.37
* PCOS*	6.37% (10/157)	16.67% (55/330)	
* Male infertility*	20.38% (32/157)	14.55% (48/330)	
* Others*	28.66% (45/157)	27.88% (92/330)	
Endometrium preparation protocol			
* Natural cycle*	49.04% (77/157)	50.60% (167/330)	0.77
* EP cycle*	50.96% (80/157)	49.40% (163/330)	
Stage of embryo transferred			
* Cleavage*	57.32% (90/157)	60.61% (200/330)	0.15
* Blastocyst*	42.68% (67/157)	39.39% (130/330)	
No. of embryos transferred	1.57 ± 0.50	1.61 ± 0.49	0.49
Endometrial thickness (mm)	11.36 ± 1.61	10.23 ± 1.71	0.01
Implantation rate	43.48% (100/230)	38.10% (208/546)	0.17
Clinical pregnancy rate	60.51% (95/157)	51.21% (169/330)	0.06
Ectopic pregnancy rate	2.11% (2/95)	2.37% (4/169)	0.84
Early Miscarriage rate	12.63% (12/95)	15.38% (26/169)	0.59

BMI, body mass index; PCOS, polycystic ovarian syndrome; EP, estrogen-progesterone.

In patients with a history of ≥ 2 failed transfer cycles, patients’ basic parameters were also comparable in the LP and FP groups (**Table 2**). Both the implantation rate (43.09% versus 33.33%, *P*=0.03) and clinical pregnancy rate (60.17% versus 46.15%, *P*=0.02) were significantly higher in the LP group than in the FP group. Interestingly, endometrial thickness was also higher in the LP group (11.27 mm vs 9.89 mm; *P* = 0.00). Similar to that from unselected patients, the ectopic pregnancy rate and early miscarriage rate were also comparable between the LP and FP groups.

**Table 2:** Basic parameters and clinical pregnancy outcomes in patients with ≥ 2 failed transfer cycles.

	Luteal phase N=118	Follicular phase N=234	*P* value
Age (year)	32.40 ± 4.88	32.31 ± 5.75	0.82
Infertility duration (year)	4.52 ± 3.43	4.85 ± 3.45	0.63
BMI (kg/m^2^)	23.12 ± 3.22	23.41 ± 3.63	0.34
Infertility diagnosis			
* Tubal factor*	25.42% (30/118)	17.09% (40/234)	0.26
* PCOS*	5.93% (7/118)	19.23% (45/234)	
* Male infertility*	19.49% (23/118)	10.68% (25/234)	
* Others*	49.15% (58/118)	52.99% (124/234)	
Endometrium preparation protocol			
* Natural cycle*	46.61% (55/118)	49.57% (116/234)	0.81
* EP cycle*	53.39% (63/118)	50.43% (118/234)	
Stage of embryo transferred			
* Cleavage*	69.49% (82/118)	73.08% (171/234)	0.24
* Blastocyst*	30.51% (36/118)	26.92% (63/234)	
No. of embryos transferred	1.69 ± 0.54	1.73 ± 0.51	0.52
Endometrial thickness (mm)	11.27 ± 1.91	9.89 ± 1.71	0.00
Implantation rate	43.09% (78/181)	33.33% (131/393)	0.03
Clinical pregnancy rate	60.17% (71/118)	46.15% (108/234)	0.02
Ectopic pregnancy rate	1.41% (1/71)	1.85% (2/108)	0.67
Early Miscarriage rate	9.86% (7/71)	19.4% (21/108)	0.10

BMI, body mass index; PCOS, polycystic ovarian syndrome; EP, estrogen-progesterone.

As shown in **Table 3**, multivariate logistic regression analysis was performed to explore factors associated with clinical pregnancy in unselected patients and patients with ≥ 2 failed transfer cycles after adjusting for confounding factors. In the unselected group, only female age was an independent factor (aOR=0.82, 90% CI: 0.78-0.86, *P*=0.00). However, in patients with ≥ 2 failed transfer cycles, apart from female age, endometrial injury in the LP group was significantly associated with the clinical pregnancy rate (aOR=2.05, 90% CI:1.22-3.47, *P*=0.01).

**Table 3:** Factors associated with clinical pregnancy rate in unselected patients and patients with ≥ 2 failed transfer cycles.

	Unselected patients		Patients with ≥ 2 failed cycles	
	aOR (95% CI)	P	aOR (95% CI)	P
Age (year)	0.82 (0.78-0.86)	0.00	0.85 (0.80-0.90)	0.00
Endometrial thickness (mm)	1.03 (0.92-1.07)	0.44	1.04 (0.84-1.04)	0.51
Protocols				
* Natural vs. EP*	0.96 (0.88-1.06)	0.46	0.97 (0.91-1.05)	0.48
Stage of embryos				
* Blastocyst vs. cleavage*	1.05 (0.85-1.07)	0.28	1.01 (0.92-1.03)	0.67
Time of endometrial injury				
* Luteal phase vs. follicular phase*	1.25 (0.95-1.65)	0.07	2.05 (1.22-3.47)	0.01

EP, estrogen-progesterone; aOR, adjusted odds ratio; CI, confidence interval.

## Discussion

Currently, IVF/ICSI-ET is an effective technology to treat infertility, but the embryo implantation rate is still approximately 30%-40%. Improving pregnancy outcomes of IVF/ICSI is a substantial challenge for reproductive clinicians.

Embryo implantation, which is the first step for a successful pregnancy, includes embryo localization, adhesion, and then invasion of the endometrium. Many scholars have advocated that endometrial injury can stimulate the inflammatory response, adjust gene expression, and thus improve endometrial receptivity. As early as 2003, Barash et al. reported that local endometrial injury before embryo transfer could significantly improve the clinical pregnancy rate and live birth rate (11). In 2007, Raziel et al. reported that in patients with high-order implantation failure (≥ 4 IVF trials and ≥ 12 transferred embryos), local injury to the endometrium prior to controlled ovarian stimulation improved implantation rates and pregnancy outcomes (12). In addition, a meta-analysis including 4 randomized studies and 3 observational studies showed that in patients with repeated implantation failure, endometrial injury also significantly improved pregnancy outcomes (13). More recently, another meta-analysis including 10 studies with 1468 participants showed that both a higher live birth rate (RR 1.38, 95% CI 1.05-1.80) and clinical pregnancy rate (RR 1.34, 95% CI 1.07-1.67) were observed in patients with endometrial injury than in controls (14).

However, unlike the previous studies mentioned above, in which all participants had failed implantation cycles, other studies have also investigated the impact of endometrial injury on pregnancy outcomes in unelected patients. In 2014, a randomized controlled trial including 300 unselected subfertile women showed that endometrial injury performed in the mid-luteal phase did not change pregnancy outcomes (6). Moreover, another RCT in 2017 that included an unselected group of patients also showed that there was no significant difference in the implantation rate or live birth rate between patients with or without local endometrial injury (9). In 2019, a multicenter RCT published in The New England Journal of Medicine including 1364 women showed that endometrial injury did not result in a higher live birth rate than no intervention among women undergoing IVF (15).

Several reasons may explain the inconsistency among these studies. First, it is obvious that the type of patients (unselected patients or patients with repeated implantation failure) included is important (16). Our results also showed that the pregnancy outcomes in unselected patients were comparable between the LP and FP groups at first. However, in the subgroup (patients with a history of ≥ 2 failed transfer cycles) analysis, it was interesting to find that endometrial injury in the LP significantly improved pregnancy outcomes in this group of patients. Then, we also noticed that the timing of endometrial injury may also have an impact on pregnancy outcomes. In 2020, data showed that there was insufficient evidence to support the use of endometrial injury in the follicular phase in intrauterine insemination treatment cycles (17). Another RCT demonstrated that endometrial injury in the luteal phase before ovarian stimulation significantly enhanced the clinical pregnancy rate in women with three or more prior implantation failures (18).

Then, when is the proper time to perform endometrial injury? Recently, a randomized study directly compared the effect of endometrial injury between the proliferative vs. luteal phase on unselected women. They found that there was no significant difference in the clinical outcomes between endometrial injury in the proliferative phase (38 patients) and injury in the luteal phase (32 patients) (9). However, our data were in contrast with those from this small sample-sized study. In our study, a total of 487 patients were included, which makes the conclusion more convincing.

Several possible mechanisms by which local endometrial injury improves pregnancy outcome have also been discussed. First, the part of the endometrium damaged due to local injury recruits immune cells, which can be further differentiated into macrophages or dendritic cells and may promote implantation (19). Second, implantation-related genes were observed to be highly expressed in women after endometrial injury when compared with controls. Moreover, after endometrial injury, a series of cytokines and growth factors are secreted to induce wound healing, such as leukemia inhibitory factor (LIF), and endothelial growth factor (VEGF), which can facilitate embryo implantation. Compared with the follicular phase, the luteal phase is characterized by a large content of growth factors, cytokines and immune cells in the endometrium (20). Thus, it is more reasonable that endometrial injury performed in the luteal phase can activate more cytokines and eventually result in better pregnancy outcomes eventually. In addition, we noticed that endometrial thickness was significantly improved in both unselected patients and patients with ≥ 2 failed transfer cycles. It is known that measuring endometrial thickness is a noninvasive method to predict endometrial receptivity in IVF cycles. In addition, data from our center and a large-sample meta-analysis also confirmed that endometrial thickness was positively associated with pregnancy outcomes in patients with embryo transfer (21–23). Thus, in our next work, we plan to collect endometrial tissue after endometrial injury in both the LP and FP and to detect endometrial receptivity-associated genes or cytokines to confirm this hypothesis.

Even though our study was one of the very few studies comparing the different effects of endometrial injury in the LP and FP with a larger sample size, there were indeed several limitations. First, the first endometrial injury was performed in our center in 2009, and it has been a routine practice for patients with repeated IVF cycles as we observed an improvement in pregnancy outcomes after endometrial injury. However, it would be better to include a sham group. In addition, this was a prospective cohort study. We tried to control for other confounding factors, such as stage of embryo transferred and the endometrial preparation protocol. Moreover, there were only 118 patients with ≥ 2 failed transfer cycles in the luteal phase group. Thus, well-designed RCTs with more patients are needed in the future.

In summary, our study with a larger sample showed that endometrial injury, a slightly traumatic intervention procedure that causes little harm to patients, significantly improves pregnancy outcomes when performed in the luteal phase compared with the follicular phase in a particular group of patients with a history of ≥ 2 failed transfer cycles but not in unselected patients.

## Data availability statement

The original contributions presented in the study are included in the article/supplementary material. Further inquiries can be directed to the corresponding author.

## Ethics statement

The studies involving human participants were reviewed and approved by Medical Ethics Committee Board of First Affiliated Hospital of Zhengzhou University. The patients/participants provided their written informed consent to participate in this study.

## Author contributions

YW and ZB contributed to the conception, design, acquisition and interpretation of data, and drafting of the manuscript. LH supervised the study. All authors contributed to the article and approved the submitted version.

## Funding

This study was supported by the National Key R&D Program of China (Grant No. 2019YFA0110900), National Natural Science Foundation of China (Grant No. 82171658), Henan Province Medical Science and Technique R&D Program (Grant No. SBGJ202102094) and the Strategic Collaborative Research Program of the Ferring Institute of Reproductive Medicine, Ferring Pharmaceuticals and Chinese Academy of Sciences (Grant No. FIRMD200501).This study was supported by the National Key R&D Program of China (Grant No. 2019YFA0110900), National Natural Science Foundation of China (Grant No. 82171658), Henan Province Medical Science and Technique R&D Program (Grant No. SBGJ202102094) and the Strategic Collaborative Research Program of the Ferring Institute of Reproductive Medicine, Ferring Pharmaceuticals and Chinese Academy of Sciences (Grant No. FIRMD200501).

## Acknowledgments

The authors would like to thank all the staff from Reproductive Medical Center, First Affiliated Hospital of Zhengzhou University.

## Conflict of interest

The authors declare that the research was conducted in the absence of any commercial or financial relationships that could be construed as a potential conflict of interest.

## Publisher’s note

All claims expressed in this article are solely those of the authors and do not necessarily represent those of their affiliated organizations, or those of the publisher, the editors and the reviewers. Any product that may be evaluated in this article, or claim that may be made by its manufacturer, is not guaranteed or endorsed by the publisher.
